# Mapping the potential distribution of the principal vector of Crimean-Congo haemorrhagic fever virus *Hyalomma marginatum* in the Old World

**DOI:** 10.1371/journal.pntd.0010855

**Published:** 2023-11-27

**Authors:** Seyma S. Celina, Jiří Černý, Abdallah M. Samy

**Affiliations:** 1 Center for Infectious Animal Diseases, Faculty of Tropical AgriSciences, Czech University of Life Sciences Prague, Czech Republic; 2 Entomology Department, Faculty of Science, Ain Shams University, Abbassia, Cairo, Egypt; 3 Medical Ain Shams Research Institute (MASRI), Faculty of Medicine, Ain Shams University, Cairo, Egypt; University of Texas Medical Branch / Galveston National Laboratory, UNITED STATES

## Abstract

Crimean-Congo haemorrhagic fever (CCHF) is the most widely distributed tick-borne viral disease in humans and is caused by the Crimean-Congo haemorrhagic fever virus (CCHFV). The virus has a broader distribution, expanding from western China and South Asia to the Middle East, southeast Europe, and Africa. The historical known distribution of the CCHFV vector *Hyalomma marginatum* in Europe includes most of the Mediterranean and the Balkan countries, Ukraine, and southern Russia. Further expansion of its potential distribution may have occurred in and out of the Mediterranean region. This study updated the distributional map of the principal vector of CCHFV, *H*. *marginatum*, in the Old World using an ecological niche modeling approach based on occurrence records from the Global Biodiversity Information Facility (GBIF) and a set of covariates. The model predicted higher suitability of *H*. *marginatum* occurrences in diverse regions of Africa and Asia. Furthermore, the model estimated the environmental suitability of *H*. *marginatum* across Europe. On a continental scale, the model anticipated a widespread potential distribution encompassing the southern, western, central, and eastern parts of Europe, reaching as far north as the southern regions of Scandinavian countries. The distribution of *H*. *marginatum* also covered countries across Central Europe where the species is not autochthonous. All models were statistically robust and performed better than random expectations (p < 0.001). Based on the model results, climatic conditions could hamper the successful overwintering of *H*. *marginatum* and their survival as adults in many regions of the Old World. Regular updates of the models are still required to continually assess the areas at risk using up-to-date occurrence and climatic data in present-day and future conditions.

## Author summary

*Hyalomma marginatum* is a vector of numerous highly important human and animal pathogens. It is the principal vector of the Crimean-Congo haemorrhagic fever virus (CCHFV) in Europe. This study updated the potential distribution of *H*. *marginatum* in the Old World. The model predicted a high suitability for *H*. *marginatum* in diverse regions of Africa, along with broader areas in Asia displaying medium to high suitability. The model predicted widespread potential distributions of *H*. *marginatum* on the continental European scale, encompassing southern, western, central, and eastern regions and even extending northward to the south of Scandinavian countries. *Hyalomma marginatum* populations have not yet been established in any country in Central Europe. However, previous studies have reported sporadic records of the species across all countries within the region. Given the frequent occurrence of immature stages of *H*. *marginatum* on migratory birds heading northward to temperate Europe, it is crucial to monitor the northward spread and establishment of permanent populations of this species. Our ecological niche model for Central Europe predicted its distribution across all countries within the region, emphasizing the need for ongoing monitoring and surveillance to detect any potential changes in the distribution and establishment of *H*. *marginatum* populations. The current prediction of *H*. *marginatum* can contribute to our understanding of the disease risk areas associated with this vector.

## Introduction

Crimean-Congo haemorrhagic fever (CCHF) is the most widely distributed tick-borne viral disease in humans, extending from western China, South Asia, and the Middle East to the southeast of Europe and Africa [1]. CCHF is caused by the Crimean-Congo haemorrhagic fever virus (CCHFV). CCHFV is an emerging arbovirus associated with high fatality rates, reaching up to 40% [1–4]. CCHFV additionally ranks among the deadliest human pathogens in Africa and Eurasia [5].

*Hyalomma marginatum* sensu lato is a complex species that includes *H*. *marginatum* sensu stricto, *Hyalomma rufipes*, and other closely related species [6]. In Europe, *H*. *marginatum* remains the main vector of CCHFV. *Hyalomma marginatum* has a veterinary and public health importance, particularly if this species can transmit various tick-borne pathogens in humans and animals other than CCHFV, such as Spotted Fever rickettsia to humans, *Anaplasma* species to animals, *Babesia caballi* and *Theileria equi* (piroplasmosis) to horses, and *Theileria annulata* (tropical theileriosis) to bovines [7–10]. This is one of the tick species whose distribution and expansion are closely monitored by the European Center for Disease Prevention and Control (ECDC) because of its major medical importance [11].

Unlike *Ixodes* ticks which adopt an ambush strategy, *H*. *marginatum* actively seeks hosts using an active locomotory hunting strategy [12]. Upon spotting a host by sensing certain signals such as vibration, visual objects, carbon dioxide, ammonia, or body heat, *H*. *marginatum* can run rapidly several meters across the ground to attack the host. Therefore, they are known as “hunter ticks”. *Hyalomma marginatum* demonstrates adaptability to diverse abiotic conditions although it prefers localities with high summer temperatures [13]. As a two-host tick, immature stages feed on the same individual animal (e.g., a small mammal like hares, hedgehogs, and rodents) or a ground-dwelling bird, whereas adults prefer larger hosts such as cattle, horses, or occasionally humans [13]. Large domestic mammals play an important role in the biology of *H*. *marginatum* and the transmission of *Hyalomma*-borne pathogens. These mammals serve as hosts, supporting a high tick load, and consequently bringing *H*. *marginatum* into close proximity with agricultural workers. In addition, livestock can directly expose humans to *Hyalomma*-borne pathogens via infected blood or crushing of engorged ticks on the animals during slaughter [14–16]. Moreover, migratory birds may introduce infected *H*. *marginatum* to new regions by carrying immature ticks during feeding [13].

*Hyalomma marginatum* is widely distributed across several African countries, particularly in North Africa and the Sahel region. It can be found in Morocco, Algeria, Tunisia, Libya, Egypt, Sudan, Chad, Ethiopia, Niger, Mali, Mauritania, and Senegal. In addition to Africa, *H*. *marginatum* is prevalent in various parts of Asia, including the Middle East, India, and Caucasus. The historical known distribution of *H*. *marginatum* in Europe includes most of the Mediterranean and Balkan countries, Ukraine, and southern Russia [6]. Further expansion of its potential distribution may have occurred in and out of the Mediterranean region. The wide dispersal of *H*. *marginatum* reflects its tolerance to diverse environments, including savannah, steppe, and scrubland hill and valley biotypes [13].

*Hyalomma marginatum* was first detected in southern Germany in 2007 in Central Europe [17]. Subsequent observations have confirmed its presence in other central European countries, including Hungary in 2009 [18], Austria in 2018 [19], and the Czech Republic in 2018–2019 [20]. Typically, permanent populations of *H*. *marginatum* are limited to warmer areas of the Mediterranean basin in Europe. Permanent populations of *H*. *marginatum* in Central Europe likely to have occurred owing to ongoing climatic changes. The establishment of these populations and the northern spread of *H*. *marginatum* are possibly anticipated due to the passive transportation of immature stages by migratory birds flying to temperate Europe [21,22]. During the spring migration of birds from the southern to the northern regions, *H*. *marginatum* is possibly introduced to Central Europe [23–26]. The expansion of *H*. *marginatum* into new geographic areas raises concerns about the possible emergence of CCHFV in these novel invaded regions. The distribution of CCHFV is related to the distributional potential of its vector species. The presence of the virus, along with its vectors, reservoirs, and amplifying hosts, play a crucial role in the emergence of CCHF under suitable environmental conditions [27]. Climate change, transportation of immature ticks through international animal trade, and migratory birds significantly impact the distributional potential of *H*. *marginatum* by allowing the emergence of CCHF in new geographic regions such as Central Europe. Additionally, socioeconomic factors such as human migration and settlement, as well as increasing human populations, may also influence the emergence of various zoonotic tick-borne diseases [28]. Considering these factors, we hypothesize that several abiotic, biotic, and socioeconomic factors may affect the ability of *H*. *marginatum* to colonize European areas. Here, we assess the current potential distribution of *H*. *marginatum*, the principal vector of CCHFV, across the Old World, with a specific emphasis on Europe and a particular focus on Central Europe using an ecological niche modeling (ENM) approach. Thus, we aimed to derive detailed predictions to assess the potential invasion of new areas by *H*. *marginatum*.

## Materials and methods

### Occurrence records

The occurrence records of *H*. *marginatum* were obtained from the Global Biodiversity Information Facility (GBIF; www.gbif.org). We meticulously reviewed records for synonymous species of *H*. *marginatum*, including *Hyalomma plumbeum* Panzer, 1795, and *Hyalomma savignyi* Gervais, 1844, while carefully distinguishing them from *Hyalomma rufipes*. Only occurrence records corresponding to established populations of *H*. *marginatum* were considered in this study. These datasets were subjected to several data cleaning steps; 1) dataset was cleaned by removing duplicate records to reduce possible biases in estimating ecological niche models [29], and 2) occurrence records were filtered based on a distance filter of ≤2.5′ (≈ 5 km) using SDMtoolbox 2.4 [30] in ArcGIS 10.7.1 (Environmental Systems Research Institute (ESRI), Redlands, CA). This distance-based thinning approach retains a single unique record within each pixel, preventing overprediction at specific locations or regions. The dataset was randomly partitioned into two portions using Hawth’s Tools [31] available in ArcGIS 10.7.1: 75% for model calibration, and 25% for internal evaluation of model predictions.

### Covariate variables

Several sets of environmental variables were used as independent variables in our model to characterize environmental variations across the calibration and projection areas. These variables were defined as potential drivers that limit the distributional potential of the vector *H*. *marginatum* [32]. These variables included satellite data from WorldGrids [33], which represented daytime and nighttime land surface temperature (LST), enhanced vegetation index (EVI), and topographic wetness index (TWI). EVI data was included due to its significant role in shaping the ecological niches of tick vectors, particularly immature stages which live on vegetation [32]. EVI is also considered an important factor in reflecting soil moisture’s availability for larvae and nymphs [34,35]. Humidity and aridity are also important factors, considering their major roles in completing the life cycle of *H*. *marginatum* ticks. Effects of abiotic factors such as temperature and humidity on the distribution of *H*. *marginatum* have been previously described and used in modeling diverse tick vector species [36,37]. We also used data summarizing climate variables from CHELSA database (http://chelsa-climate.org/). CHELSA data includes 19 bioclimatic variables originally derived from monthly temperature and rainfall values collected from weather stations between 1981–2010 [38]. Bioclimatic variables 8–9 and 18–19 were excluded from the analysis to avoid problems deriving from odd spatial artifacts [39].

Climatic and socioeconomic factors influence tick populations; socioeconomic factors exert indirect effects through human activities and land-use practices. It is noteworthy that tick vectors are not confined to natural habitats alone but have successfully adapted to urban environments. In recent years, urban areas have emerged as significant sites for the expansion of tick-borne diseases within regions where these diseases are endemic [40]. Urban areas have characteristic dense human populations and offer suitable hosts and favorable conditions for tick survival. Several studies conducted in urbanized regions, including Finland, Hungary, Slovakia, Poland, and France, have revealed well-established tick populations and a comparable or even higher prevalence of tick-borne pathogens compared with endemic areas [18,41–44]. Human population, migration, and transportation play significant roles in shaping tick distribution worldwide [28]. Higher human density increases the availability of tick hosts, whereas migration introduces ticks and diseases to new regions. Transportation networks additionally facilitate tick movement and allow their invasion into novel areas. To comprehensively examine the interplay among climate, socioeconomic factors, and tick vectors, our model included anthropogenic data. Anthropogenic data consisted of data on human population density from 2015 to 2020, nighttime lights, and accessibility via transportation that may play a role in the distribution of *H*. *marginatum* ticks. Population density grids were obtained from the Gridded Population of the World, version 4 (GPWv4) available at http://beta.sedac.ciesin.columbia.edu/data/collection/gpw-v4 [45]. Nighttime satellite imagery was obtained from the NOAA-Defense Meteorological Satellite Program (http://ngdc.noaa.gov/eog/dmsp/downloadV4composites.html) and was used as a proxy for poverty estimates [46,47]. Accessibility was summarized in terms of travel time by land or sea [48], as connectivity between population sites is an important variable in estimating the potential distributions of disease vectors and emerging diseases [29,49]. This layer was developed by the European Commission and World Bank (http://forobs.jrc.ec.europa.eu/products/gam/download.php). All environmental and socioeconomic variables were resampled to a spatial resolution of 5 km. The importance of the variables was evaluated using the Jackknife function in Maxent. The final sets of variables used in our analysis were as follows: Set 1 (15 bioclimatic variables from CHELSA); Set 2 (12 variables selected based on Jackknife test in Maxent); Set 3 (a combination of 15 bioclimatic variables and satellite data summarizing daytime and nighttime LST, EVI, TWI, population density, nighttime satellite imagery, and accessibility); and Set 4 (only satellite data summarizing daytime and nighttime LST, EVI, TWI, population density, nighttime satellite imagery, and accessibility) (**Table 1**).

**Table 1:** Settings and variables used for the construction of the ecological niche modeling for *Hyalomma marginatum*.

Covariate Variables	Code	Candidate sets of environmental variables of *H*. *marginatum* model			
		Set1	Set2	Set3	Set4
Annual Mean Temperature	Bio1	x	x	x	
Mean Diurnal Range	Bio2	x	x	x	
Isothermality	Bio3	x	x	x	
Temperature Seasonality	Bio4	x	x	x	
Maximum Temperature of Warmest Month	Bio5	x	x	x	
Minimum Temperature of Coldest Month	Bio6	x	x	x	
Temperature Annual Range	Bio7	x	x	x	
Mean Temperature of Warmest Quarter	Bio10	x	x	x	
Mean Temperature of Coldest Quarter	Bio11	x	x	x	
Annual Precipitation	Bio12	x		x	
Precipitation of Wettest Month	Bio13	x		x	
Precipitation of Driest Month	Bio14	x	x	x	
Precipitation Seasonality	Bio15	x	x	x	
Precipitation of Wettest Quarter	Bio16	x		x	
Precipitation of Driest Quarter	Bio17	x	x	x	
Land Surface Temperature	LST			x	x
Enhanced Vegetation Index	EVI			x	x
Topographic Wetness Index	TWI			x	x
Population density	GPW			x	x
Nighttime satellite imagery	NTL			x	x
Accessibility	-			x	x

### Accessible area (“*M*”)

The accessible area ***“M”*** is a crucial component of the biotic, abiotic, and movement (***BAM***) diagram [50]. It defines the key parameters for constructing an ecological niche model for the species in question. Accessible area ***“M”*** indicates the areas that the species explored and had access to over relevant periods of the species’ history [51]. The delimitation of the *H*. *marginatum* accessible area ***“M”*** was estimated using the *grinnell* package in R [52]. This method simulates dispersal and accessibility based on niche estimations. The *grinnell* package uses a combination of clean occurrence records of the target species and a set of covariates as inputs to estimate the niche.

### Ecological Niche Modeling

We constructed ENM using the maximum entropy algorithm implemented in the *kuenm* R package [53]. While there are various model algorithms available for estimating niche models, such as generalized linear models (GLM), generalized additive models (GAM), and boosted regression trees (BRT), each designed for different types of distribution data and modeling purposes [54], we selected Maxent because of its efficiency in handling complex interactions between response and predictor variables [55]. A total of 1972 candidate models (i.e., these models refer to the different model configurations or combinations of predictor variables considered during modeling) were built with four distinct sets of environmental and socioeconomic variables. These models were additionally constructed based on parameters reflecting all combinations of 17 regularization multiplier settings (0.1–1 with intervals of 0.1, 2–6 with intervals of 1, and 8 and 10; i.e., these settings determine the level of complexity or smoothness of the model), and 29 possible combinations of 5 feature classes (linear = l, quadratic = q, product = p, threshold = t, and hinge = h).

The best candidate model was selected based on three different criteria: 1) significance, 2) performance, and 3) the Akaike information criteria (AIC): AICc, delta AICc, and AICc weights. Performance was measured using the omission rate, which is a threshold that considers an estimate of the likely amount of error among occurrence data and thus removes 5% of occurrences with the lowest suitability values (E  =  5%) [56]. Models were selected with delta AICc ≤ 2 from those that were statistically significant and had omission rates below 5%. We followed the criteria from the original *kuenm* study [53] to select and evaluate the final model. We created the final model of *H*. *marginatum* using 10 replicates by bootstrap, with logistic, product, and hinge outputs. These models were finally transferred from the accessible area ***“M”*** to the projection area ***“G”***.

### Extrapolation Risk of *H*. *marginatum*

This analysis identified the areas with extrapolation risk based on a mobility-oriented parity (MOP) approach to compare the environmental breadth of predictors within ***“M”*** (10% reference points sampled) with that in the projection area. Mobility-oriented parity analysis was performed using the MOP function [57] available in *kuenm* package in R. The risk of extrapolation analysis calculates multivariate environmental distances between projection area ***“G”*** and the nearest portion of the calibration region to identify areas that have a condition of strict or combinational extrapolation.

### Independent evaluation

We used a set of additional independent records for the final model evaluation. These records were retrieved from a previous study [32] for additional evaluation of model performance to assess its ability to anticipate risk in unsampled areas. We cleaned the dataset to keep only unique records and double-checked the records to remove any source of overlap with the data used in the model training and testing (i.e., the occurrence records employed in this evaluation differ from those used for model calibration, specifically referring to occurrences distinct from the records obtained from GBIF for model calibration). These records were tested for successful predictions based on a binary model indicating areas where the species was present (suitable) or absent (unsuitable). The continuous model was converted to a binary map based on a threshold value. The threshold value was determined based on a maximum allowable omission error rate of 5%, accounting for potential errors in covariate values within 5% of the occurrences. Subsequently, a one-tailed cumulative binomial probability test was used to assess the probability of obtaining the observed level of correct predictions by chance alone, given the background expectation of correct predictions determined by the proportional coverage of the study area by regions of predicted suitability.

## Results

**Fig 1:**
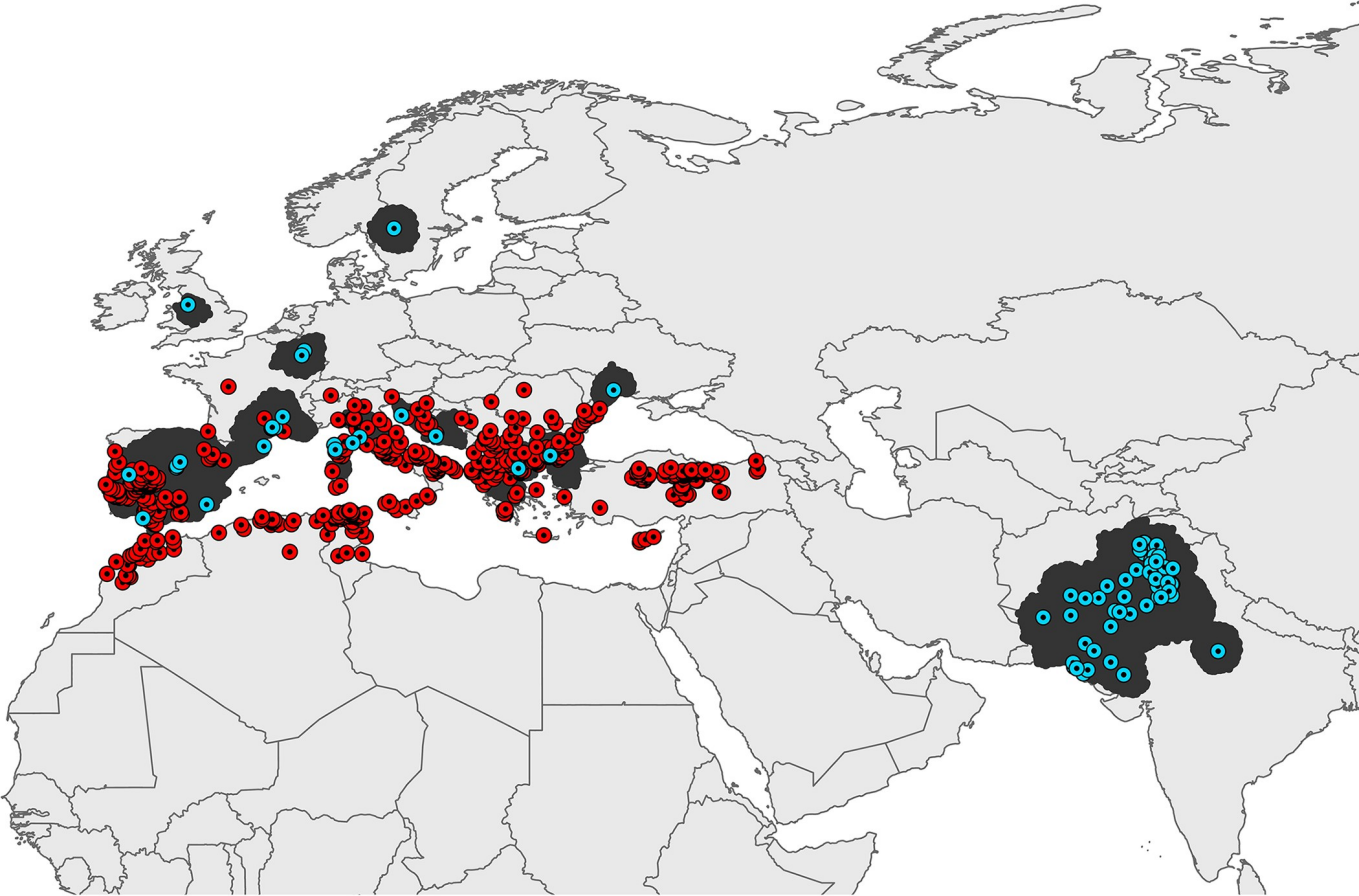
*Hyalomma marginatum* occurrence records used in model calibration and final model evaluation across Europe, North Africa, Western and South-Central Asia. The blue dotted circles represent the occurrence records of *H*. *marginatum* collected from GBIF used for model calibration across the ***“M”*** area. The red dotted circles represent the retrieved occurrence records of *H*. *marginatum* from the literature used for the final model evaluation. Dark grey polygons represent the accessible areas (***“M”***) where the *H*. *marginatum* model was calibrated. The base layer of all the maps (from Fig 1 to Fig 5) is extracted from ArcGIS Hub developed by ESRI. https://arc-gis-hub-home-arcgishub.hub.arcgis.com/datasets/esri::world-continents/explore?location=0.736373%2C36.668919%2C2.65.

The final dataset used for calibration, derived from GBIF, comprised 95 unique occurrence records after removing duplicated and redundant occurrence records, and the diverse cleaning steps (**Fig 1**). Of the 1972 candidate models, 1846 models were statistically significant. After applying the three selection criteria, a single model successfully met all requirements and was designated as the best candidate model, as evidenced by its performance (**Table 2**). The optimal model used Set4 of the satellite data, summarizing daytime and nighttime land surface temperature, EVI, TWI, population density, nighttime light, and accessibility.

**Table 2:** The best candidate model for the construction of the ecological niche modeling for *Hyalomma marginatum*. Model performance under optimal parameters using sets of environmental predictors (SEP), statistically significant models (SSM), best candidate models (BCM), regularization multiplier (RM), features classes (FC), mean Area Under the Curve ratio (AUC.r), partial Receiver Operating Characteristic (p.ROC), omission rate 5% (O.rate 5%), Akaike information criterion corrected (AICc), delta Akaike information criterion corrected (ΔAICc), Akaike information criterion corrected weight (AICc.W), number of parameters (#; summarizes the combination of environmental variables, multiple regularizations, and features other than 0 that provide information for the construction of the model based on lambdas—lambda refers to counting all parameters with a nonzero weight in a Maxent-generated text file), and environmental variables of Set4 tested during calibration of *Hyalomma marginatum* model.

SEP	SSM	BCM	RM	FC	AUC.r	p.ROC	O.rate 5%	AICc	ΔAICc	AICc.W	#
Set4	1846	1	3.0	lph	1.21	0.00	0.05	1960.75	0.00	1.00	15
Set4											
		Daytime and Nighttime Land Surface Temperature, Enhanced Vegetation Index, Topographic Wetness Index, Population density, Nighttime satellite imagery, and Accessibility									

* q = quadratic; t = threshold; h = hinge; p = product.

**Fig 2:**
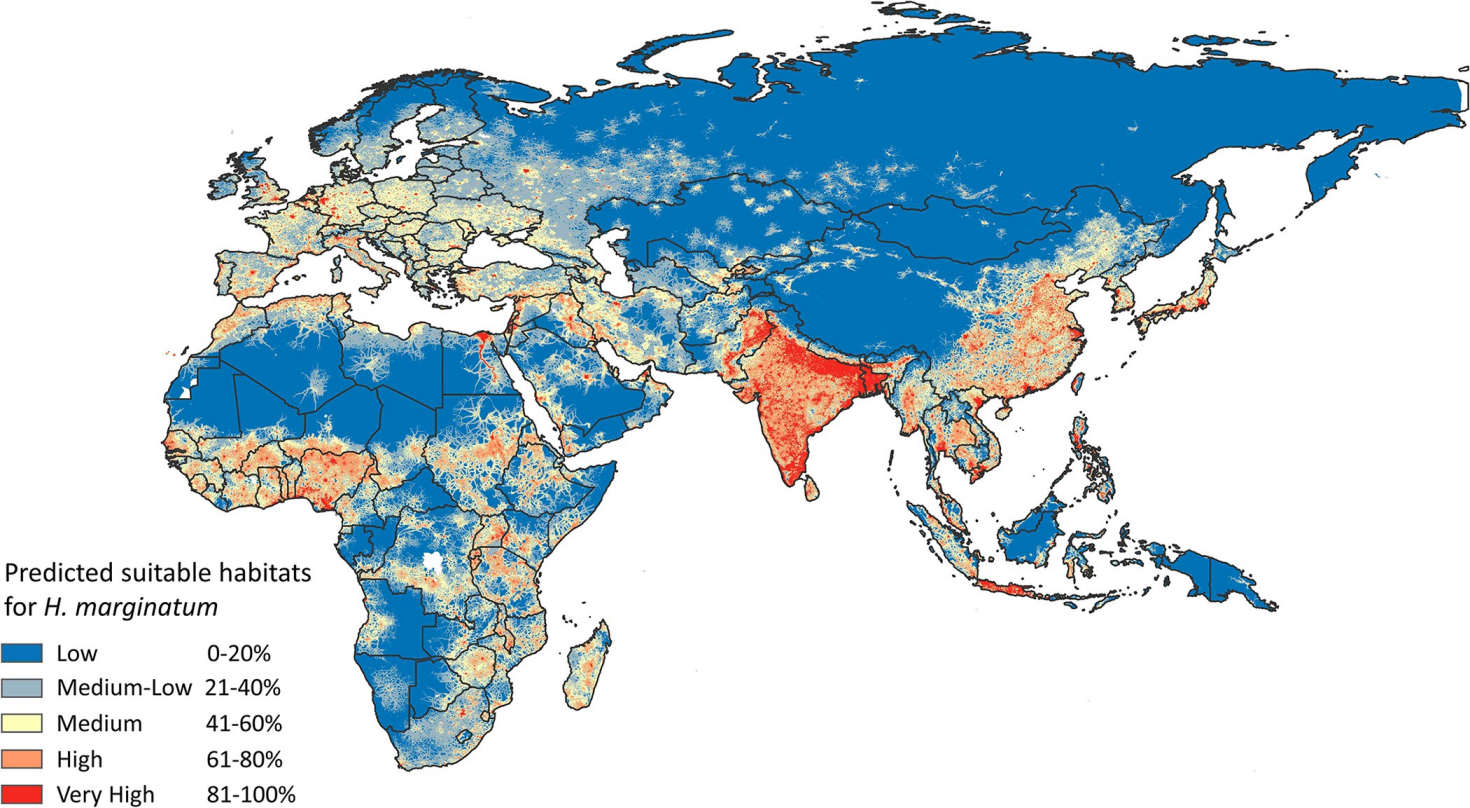
Predicted potential geographic distribution of Crimean-Congo haemorrhagic fever vector *Hyalomma marginatum* on a global scale. Red colors indicate highest habitat suitability and blue lowest suitability. The base layer of all the maps (from Fig 1 to Fig 5) is extracted from ArcGIS Hub developed by ESRI. https://arc-gis-hub-home-arcgishub.hub.arcgis.com/datasets/esri::world-continents/explore?location=0.736373%2C36.668919%2C2.65.

High to very high suitability of *H*. *marginatum* occurrence was observed in various regions of Africa, including the northern parts of North Africa (Morocco, Tunisia, and Egypt), West and East Africa, central parts of Central Africa, and eastern parts of Southern Africa (**Fig 2**). In Asia, the model indicated medium to high suitability in expansive areas of the continent, encompassing India, Pakistan, Bangladesh, and parts of China (**Fig 2**).

**Fig 3:**
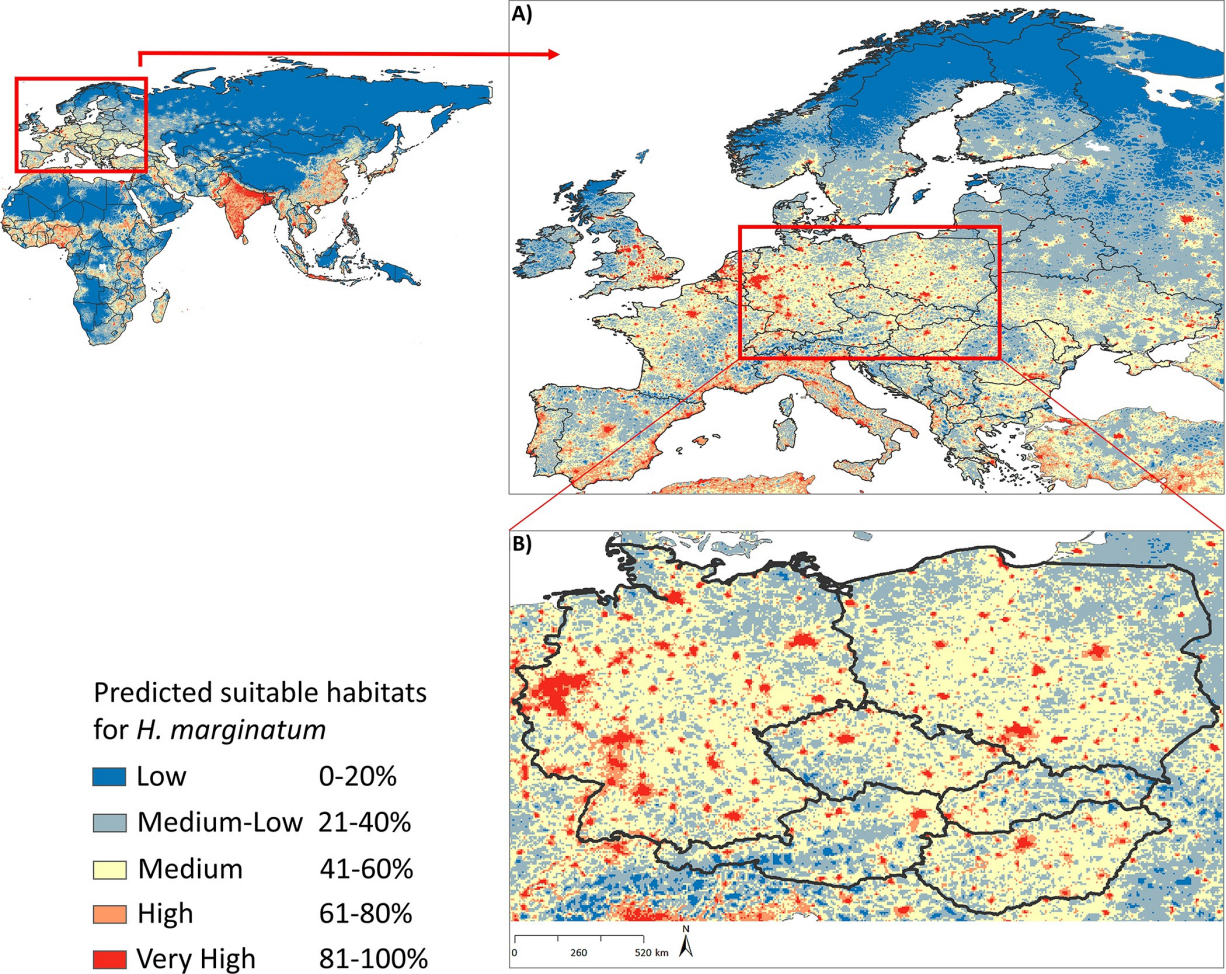
Predicted potential distribution of Crimean-Congo haemorrhagic fever vector *Hyalomma marginatum* on a global scale (left top), and close-ups of Europe (A) and Central Europe (B), to provide additional detail to predictions in the region. Red areas indicate modeled highest suitable conditions, and blue areas are lowest suitable conditions. The base layer of all the maps (from Fig 1 to Fig 5) is extracted from ArcGIS Hub developed by ESRI. https://arc-gis-hub-home-arcgishub.hub.arcgis.com/datasets/esri::world-continents/explore?location=0.736373%2C36.668919%2C2.65.

On a continental European scale, the model projected a widespread potential distribution of *H*. *marginatum* (**Fig 3**). Thus, the model anticipated the occurrence of *H*. *marginatum* in Southern, Western, Central, and Eastern Europe, as far north as the southern parts of Scandinavian countries (**Fig 3A**). A high probability of suitable conditions for *H*. *marginatum* in Southern Europe, especially in the Mediterranean parts of Spain, Portugal, Italy, and Greece and along the Adriatic shore, is presented. In the Balkan Peninsula, the southeastern part of the continent, wide areas of all countries were identified as highly suitable areas for *H*. *marginatum*. In Western Europe, large areas of northern and southern France, as well as the Benelux states, were predicted to be highly suitable for the distribution of tick species. Although the Scandinavian countries generally presented low suitability, the model predicted medium to high suitability in the southern regions of most Northern European countries (Sweden, Norway, Denmark, and Finland except Iceland), as well as high suitability in scattered areas in the southern parts of Northern European countries. The northeastern region of Europe primarily demonstrated low and low–medium suitability for *H*. *marginatum* distribution, with a few scattered points indicating medium to high suitability in the urban areas of Baltic countries (Estonia, Latvia, and Lithuania). In northwestern Europe, the model depicted medium suitability across all areas in the UK, with very high suitability in specific regions such as the southeast and southwest of England, the East Midlands, the West Midlands, Yorkshire, London, and the southern parts of the northwest and central parts of Northeast England. In Scotland, Ireland, and Northern Ireland, low and low–medium suitability for *H*. *marginatum* distribution predominated. Eastern Europe, including Romania, Ukraine, and Moldova, was identified as a highly suitable area, characterized by medium to very high suitability.

The ENM of *H*. *marginatum* in Central Europe provided insights into its distribution across all countries in the region (**Fig 3B**). The model indicated broader environmental suitability in Germany, Poland, Hungary, and the Czech Republic, with Austria and Slovakia following suit. Germany exhibited medium to high suitability, with the highest suitability observed in North Rhine Westphalia, Hesse, Saarland, Baden-Württemberg, Bremen, Berlin, Hamburg, and scattered areas in Bavaria, Thuringia, Saxony, and Lower Saxony. The same is true for large parts of Poland and Hungary, particularly the entire city of Budapest, which was identified as a very high suitable habitat for *H*. *marginatum* occurrence. High-risk areas occurred in eastern Austria and adjoining areas, and in the western and northern parts of Slovakia. While large areas of the Czech Republic have medium suitability, very high suitability of *H*. *marginatum* also occurred in several scattered areas.

**Fig 4:**
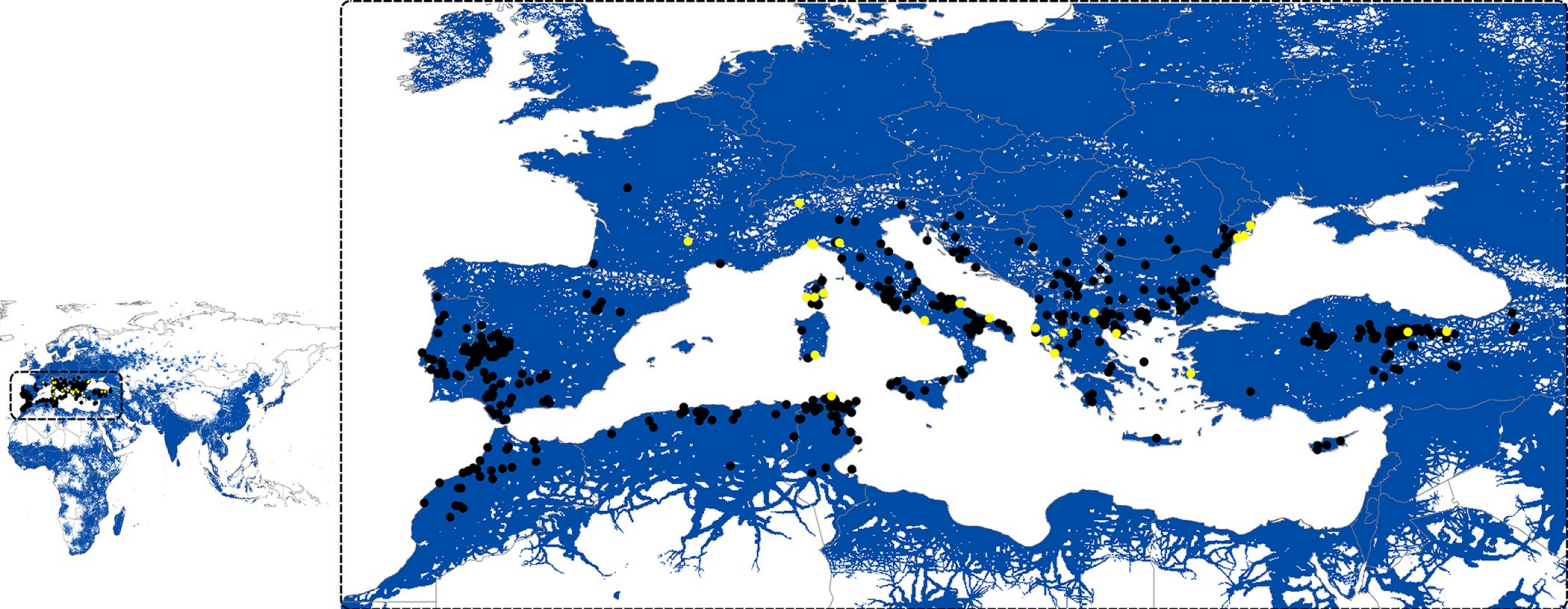
Relationship of *Hyalomma marginatum* ecological niche modeling prediction to the distribution of the independent set of *Hyalomma marginatum* occurrence records. Blue shading shows areas predicted suitable for *Hyalomma marginatum* occurrences. Black and yellow dots represent the independent records of *Hyalomma marginatum* used for the final model evaluation; black dots are records with successful prediction and yellow dots are records where the prediction is not captured by the model. The base layer of all the maps (from Fig 1 to Fig 5) is extracted from ArcGIS Hub developed by ESRI. https://arc-gis-hub-home-arcgishub.hub.arcgis.com/datasets/esri::world-continents/explore?location=0.736373%2C36.668919%2C2.65.

The final model was evaluated using independent occurrence data [32], comprising 621 points after eliminating duplicated and redundant records through various cleaning steps. The independent set of *H*. *marginatum* occurrence records were related to the *H*. *marginatum* model prediction. The model successfully predicted 588 of 621 (94.68%) independent data (**Fig 4**).

**Fig 5:**
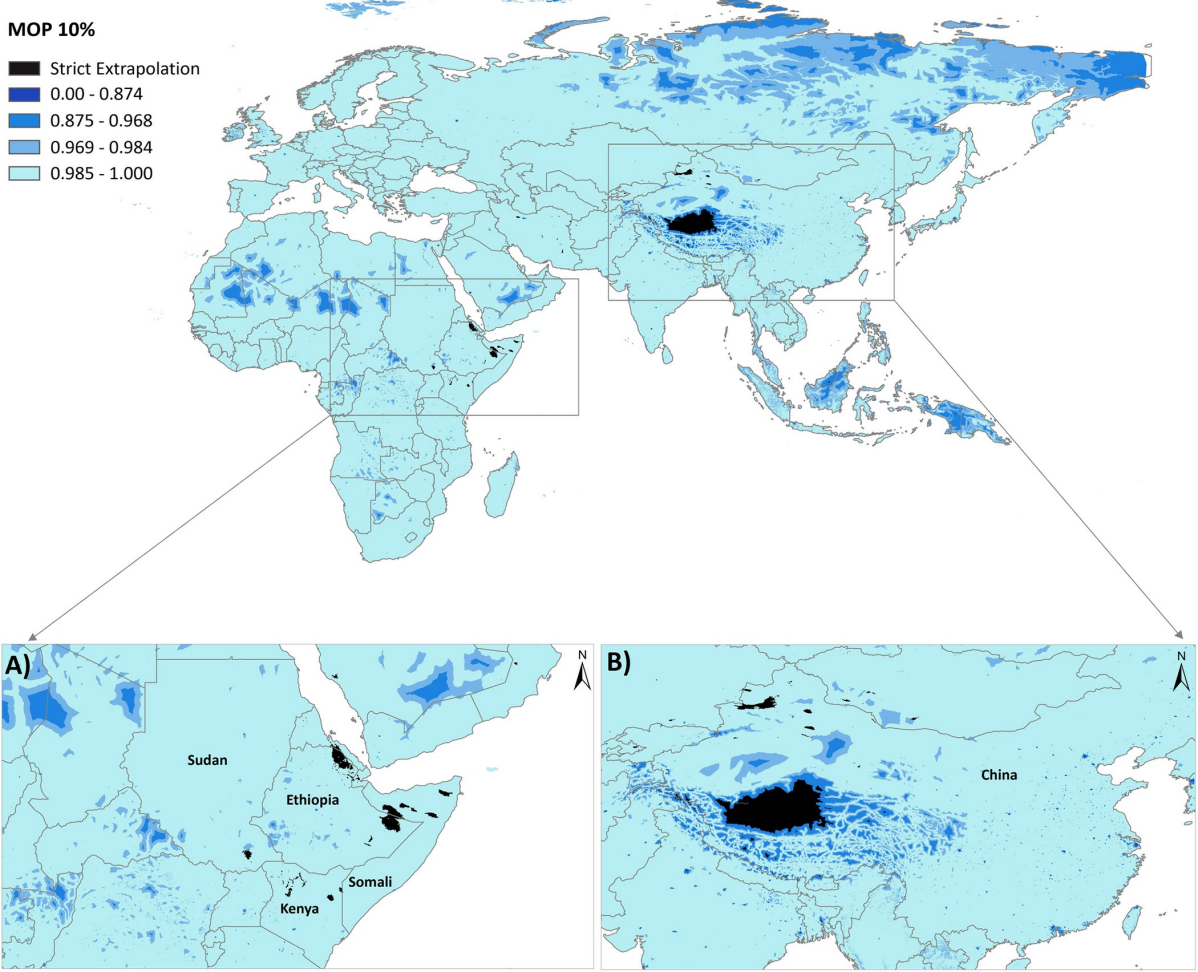
Mobility-oriented parity (MOP) 10% extrapolation risk analysis for the ecological niche model of *Hyalomma marginatum* from the calibration area *(“M”*) to a projection area (*“G”*) (top), and close-ups of East Africa (A) and Eastern Asia (B), to provide additional detail to strict extrapolations occurred in the areas. The MOP analysis indicated that areas with the most dissimilar variables conditions (i.e., where one or more covariate variables are outside the range present in the training data) were found beyond the potential distributional areas predicted by the model in the **“G”** area. Areas with the most dissimilar variables conditions display strict extrapolative areas and are represented by zero value. Other values represent levels of similarity between the calibration area and the **“G”** transfer area. The MOP raster output was reclassified into five categories; the first category represented a strict extrapolation (i.e., zero value), and the fifth category represented the highest environmental similarities between calibration and projection areas. The base layer of all the maps (from Fig 1 to Fig 5) is extracted from ArcGIS Hub developed by ESRI. https://arc-gis-hub-home-arcgishub.hub.arcgis.com/datasets/esri::world-continents/explore?location=0.736373%2C36.668919%2C2.65.

The MOP results indicated high levels of environmental similarities in all areas under the question, except Southwest China (e.g., northern parts of Tibet province), Northwest China (e.g., southern parts of Xinjiang province), and some areas in East Africa (e.g., Ethiopia, Kenya, Somalia, Sudan) where strict extrapolation occurred (**Fig 5**). Therefore, predictions on these areas should be taken with caution because they were consistently detected as areas with high extrapolation risk.

## Discussion

Ecological niche modeling has been widely implemented to predict the distribution of numerous vector-borne diseases [29,39,58–64]. These modeling studies play a crucial role in enhancing the efficiency of vector surveillance and control programs.

In this study, we provided detailed maps to identify the potential distribution of *H*. *marginatum*, principal vector of CCHFV, in the Old World, particularly in Europe, with a specific emphasis on Central Europe. This study also addresses model uncertainty under current environmental and socioeconomic conditions. These conditions highlight significant factors influencing the spread of ticks and tick-borne diseases, including climate change, vegetation patterns, human population density, poverty levels, and increased movement of animals and humans due to improved accessibility. The introduction of *Hyalomma* species to new geographical areas is likely facilitated by infested hosts resulting from human activities, the movement of domesticated and wild mammals, and migratory birds [10,65].

In Europe, *H*. *marginatum* is mostly endemic to Mediterranean countries, but isolated populations also exist in Transalpine Europe. Our study revealed extensive areas of high suitability for *H*. *marginatum* occurrence across the entire European continent. Specifically, high suitability was observed in the Mediterranean regions of Spain, Portugal, Italy, Greece, and along the Adriatic shore. Furthermore, southern, and northern France, large areas in the Netherlands, Belgium, Germany, Balkan countries, Ukraine, Crimea, scattered areas in Central Europe, and even extending into the United Kingdom remained favorable conditions for the tick species. A previous study anticipated high environmental suitability of *H*. *marginatum* in southern, southeast, and Central Europe, as well as regions in North Africa (Tunisia, Morocco, and Algeria), the Arabian Peninsula, south-central Asia, and China [66]. Our model improved the prediction and provided a more reliable and detailed map of habitat suitability for *H*. *marginatum*. Similar to the previous ecological niche model [66], the highest environmental suitability for *H*. *marginatum* was predicted across Southern Europe. In addition, we observed high suitability across all Balkan countries in southeast Europe. Interestingly, our predictions differed from those of the previous model in several aspects. We identified broader areas of high and very high suitability, particularly in Balkan countries (Albania, Croatia, Bosnia and Herzegovina, Kosovo, Montenegro, Romania, and Slovenia), Western Europe, the United Kingdom, and southern parts of Scandinavia, which were underestimated by Okely and colleagues [66]. This previous study ignored important model settings that can lead to errors in model estimation. In addition to the prediction of Okely and colleagues, previous studies have mapped the geographic distribution of *H*. *marginatum* at regional and global scales [67,68]. In comparison with these studies, our model anticipated higher suitability for the occurrence of *H*. *marginatum* in Central, Western, Eastern, and Northern Europe, contradicting their predictions of low suitability for this tick species in the same areas.

*Hyalomma marginatum* was first reported in Central Europe in Germany in 2007 [17]. Although it was recorded in other countries of Central Europe, including Hungary [18], the Czech Republic [20], and Austria [19], these observations did not represent stable populations of *H*. *marginatum* in Central Europe. *Hyalomma marginatum* ticks have ecological plasticity that can support several tolerance ranges to temperature and humidity conditions [69]. This tick species prefers dry and warm regions and is most abundant during summer. In the northern hemisphere, it becomes active in spring when temperatures rise, typically around April [70]. Immature stages are active from June to October, peaking in numbers during July and August. After a blood meal, immature ticks either drop in early summer, molt into adults during the same season, and overwinter as adults, or drop in late summer, overwinter as nymphs, and molt into adults the following spring [70]. Stable populations of *H*. *marginatum* in Europe are restricted to the warm areas of the Mediterranean basin and are absent in Central Europe, likely because of unsuitable environmental conditions. However, our model predicted its distribution across all countries in Central Europe and provided strong evidence for extensive medium to very high suitability in Germany, Poland, Hungary, and the Czech Republic, followed by Austria and Slovakia. A recent study conducted in Hungary revealed seropositivity for CCHF, indicating that Hungary could be a novel geographical region for the distribution of CCHFV [71]. Sporadic records of *H*. *marginatum* in other central European countries, apart from Hungary, indicate potential breeding habitats for this tick vector. In Austria, the first recorded occurrence of *H*. *marginatum* was an adult male tick found on a horse in 2018 [19]. Recent studies in the Czech Republic and Slovakia reported *H*. *marginatum* complex species exclusively on migratory birds [72]. In addition, a very recent study reported the collection of five adult *H*. *marginatum* ticks from horses and households in the Czech Republic [20]. Although *H*. *marginatum* does not belong to the endemic tick fauna in Germany, sporadic findings have been reported recently (e.g., 2007, 2011, 2017, and 2018) [73]. In Poland, records of *H*. *marginatum* date back to earlier times, with unfed male ticks reported in 1935 and 1943, which are currently archived in the Bytom Museum collection [74,75]. The introduction of these adult ticks to Central Europe is highly probable because they likely originated as nymphs feeding on migratory birds and molted into adults after becoming fully engorged. The environmental conditions in Central Europe offer suitable habitats for *Hyalomma* spp. to develop and locate appropriate vertebrate hosts for subsequent blood feeding. While it has been proposed that *H*. *rufipes*, another significant vector of CCHFV, overwinters in Central Europe [76], there is currently no speculation regarding the establishment of *H*. *marginatum* populations in the region.

Furthermore, our results showed medium to high environmental suitability for *H*. *marginatum* in the south of Scandinavia. In Northern Europe, documented records of *H*. *marginatum* consist solely of transported immature stages found on migratory birds during their northward spring migration. These records include instances in Denmark in 1939 and 1991 [23,77], Finland in 1962 and between 2018 and 2020 [78,79], Norway in 1994 [23], and Sweden between 1990 and 1991 [23]. Although no scientific study has reported the occurrence of *H*. *marginatum* in Norway since 1994, Hasle et al. [80] reported the presence of seven fully engorged nymphs of *H*. *rufipes* in migratory birds captured during 2003–2005. Recently, the first sightings of adult *H*. *marginatum* ticks have been documented on horses, cattle, and humans in Sweden [81]. The rapidly changing climate in Northern Europe appears to create favorable conditions for the development of immature stages of this tick species, carried by migratory birds, to reach the adult stage and potentially attach and feed on large mammals, including humans.

Appropriate climatic and biotic conditions in the regions that are depicted in our maps may provide a suitable environment for introduced *H*. *marginatum* ticks. A warming climate trend could enhance the persistence of infected *H*. *marginatum* ticks. The expansion of suitable areas has been documented in Central Europe [82]. Thus, it is possible to hypothesize that climate change may potentially lead to an increase in winter temperatures, leading to an increase in the probability of *H*. *marginatum* overwintering, and subsequently raising the risk of its establishment in various parts of central European countries. Therefore, modeling studies based on future scenarios should be implemented to better understand the impact of climate change on the dispersion of *H*. *marginatum*.

ENM results are valuable for understanding species tolerance to biotic and abiotic factors. When combined with knowledge of ecology, behaviour, and life history, they assist in selecting the most realistic predictions [83]. Our current global potential distribution prediction for this tick vector can provide insights into disease risk areas associated with this vector. However, it is essential to assess the uncertainty in model projections, particularly for decision-makers in vector and disease surveillance and public health. CCHFV, a vector-borne pathogen, requires a “One Health” approach that requires a multidisciplinary perspective encompassing animal, human, and environmental health [84]. Therefore, it is crucial to develop effective control strategies that address emerging *Hyalomma*-borne diseases within the framework of a “One Health” perspective, where a rapidly changing environment and animal health may impact human health.

In this study, we used a novel improved methodology and allowed different modeling settings to construct the habitat suitability model for *H*. *marginatum* based on calibration and evaluation of our models using several updated variables that previously were not used. Although our continental-scale predictions provide valuable insights into the distribution of *H*. *marginatum*, it is crucial to investigate the species’ interactions with microclimate and the influence of host availability on its response to prevailing climatic conditions [85].

The model effectively identified known areas where the disease is prevalent, but it has a few limitations. Notably, the abundance of occurrence records in Europe and the Indian subcontinent has probably led to wider predictions, particularly in urban areas and transportation routes. It is noteworthy to mention that human-modified landscapes, such as urban areas, have gained attention as potential habitats for ticks, contrary to the belief that they are restricted to natural habitats. Studies conducted across Europe have revealed the successful establishment of tick populations in urban areas, and the spread of tick-borne diseases has been linked to these urban hotspots [18,41–44]. The tick ability to thrive in urban environments emphasizes the significance of considering urban areas as potential hubs for tick populations.

Furthermore, areas with a low number of occurrence records driven by accessibility bias should be further investigated, especially if the model predicts a high suitability habitat for *H*. *marginatum*. However, it is essential to distinguish between two key aspects: 1) occurrence records, which represent sampling efforts in specific areas influenced by factors such as tick identification expertise or the need for intensive fieldwork to study tick-borne disease circulation, and 2) model predictions generated by applying algorithms to thinned occurrence records and covariate data. Occurrence records indicated the presence or absence of the species within the sampled areas, whereas the model provided probability values indicating pixel suitability for species presence. This pattern enables the assessment of tick suitability in unsampled areas based on available occurrence records and covariate information, which poses a significant challenge in ecological niche modeling. To ensure reliable predictions, our model underwent careful calibration, and necessary adjustments were implemented to avoid over-prediction. Host parameters were not incorporated into the model because the species’ broad host range encompassed numerous vertebrate species. Incorporating host species data poses challenges because obtaining reliable occurrence information for multiple hosts is difficult, and the optimal approach to their inclusion remains unclear [36]. Nevertheless, although *H*. *marginatum* is a generalist species, certain vertebrate hosts may be preferred within its extensive range of potential hosts [86]. However, caution should be exercised when interpreting these host preferences as they may vary across different regions [87]. Finally, the Maxent algorithm used in ecological niche modeling has a few limitations, including reliance on presence-only data that can introduce biases, potential overfitting with complex predictor variables, the risk of unreliable results when extrapolating beyond calibrated conditions, limited mechanistic understanding and sensitivity to variable selection, and the impact of spatial autocorrelation and the assumption of independence on model performance. Considering these limitations is essential when using this model.

In summary, our future studies will consider further detailed mapping of CCHF disease and *H*. *marginatum* distribution under different climate change scenarios. In addition to mapping the distribution of this tick species under different future projections, our understanding of the dynamics of the host–tick vector–virus system will be improved by further modeling studies that incorporate the effects of vertebrate hosts (different types of hosts, seasonality, and movements) as well as life traits of the tick species.
